# Effect of visually cued spatial and temporal attention on audiovisual stimuli processing: an event-related potentials study

**DOI:** 10.3389/fpsyg.2025.1591768

**Published:** 2025-05-21

**Authors:** Yang Feng, Kai Liu, Rui Zhang, Huiyuan Wang, Yulin Gao, Zhihan Xu, Jingjing Yang

**Affiliations:** ^1^School of Teacher Education, Huzhou University, Huzhou, China; ^2^Department of Psychology, Jilin University, Changchun, China; ^3^Department of Foreign Language, Ningbo University of Technology, Ningbo, Zhejiang, China; ^4^School of Artificial Intelligence, Changchun University of Science and Technology, Changchun, China

**Keywords:** spatial attention, temporal attention, audiovisual stimuli, interstimulus interval (ISI), event-related potentials (ERPs)

## Abstract

**Introduction:**

Previous studies have investigated the effect of spatial and temporal attention on visual or auditory stimulus processing. The visual and auditory information received simultaneously from different modalities must be integrated by several systems to produce coherent cognition in the brain. However, how spatial and temporal attention modulates audiovisual (AV) stimuli processing is still unclear.

**Methods:**

The aim of this study was to compare the modulatory effects of spatial attention versus temporal attention on audiovisual stimuli processing using event-related potentials (ERPs) with high temporal resolution. Spatial attention was triggered by a visual spatial cue (usually an arrow), and temporal attention was triggered by a visual temporal cue (two concentric circles).

**Results:**

Behavioral responses to audiovisual stimuli in the spatial attention condition were faster than those in the temporal attention condition, and the false alarm rate in the spatial attention condition was lower than that in the temporal attention condition. The ERP results show that the amplitude of N2 elicited by AV stimuli in the right temporal and right occipital areas in the spatial attention condition was greater than that in the temporal attention condition.

**Discussion:**

These results indicate that spatial and temporal attention have a differential effect on AV stimuli processing in the right occipitotemporal area.

## Introduction

In the real world, there is a large amount of information bombarding us. Attention can help us to selectively concentrate on one aspect of the useful information in the environment while ignoring others. Attention is distributed across time as well as space. Spatial attention refers to the allocation of attentional resources to specific spatial locations (Posner, 1980). Similarly, temporal attention refers to the allocation of attentional resources to specific moments in time, that is, selecting the most relevant information at a particular moment and prioritizing it (Griffin et al., 2001; Denison et al., 2021), which helps to improve the accuracy of recognition, detection and temporal resolution as well as the reaction time at the moment of attention (Coull, 2004; Fernández et al., 2019).

In daily life, spatial attention and temporal attention often occur simultaneously, some studies have explored whether spatial attention and temporal attention are operated by different attentional mechanisms. Previous studies have found that there is hemispheric asymmetry in spatial attention, with the right hemisphere typically showing stronger activation than the left hemisphere (DiNuzzo et al., 2022). In contrast to spatial attention, the left hemisphere shows stronger activation in temporal attention (Davranche et al., 2011). There are also studies using a simple detection task, demonstrating that endogenous temporal and spatial attention are fundamentally independent of each other but can interact under conditions of high perceptual demands (Weinbach et al., 2015; Rohenkohl et al., 2014). Therefore, temporal attention and spatial attention are two distinct attention systems, and they may have different effects on stimulus processing.

A previous neurophysiologic positron emission tomography (PET) and functional magnetic resonance imaging (fMRI) study used a visual cue-visual target paradigm to investigate the effects of spatial and temporal attention on visual stimulus processing (Coull and Nobre, 1998). This study found a partial overlap between the neural systems involved in spatial and temporal attention. Specifically, hemispheric asymmetries revealed that spatial attention is preferentially processed by the right cortex, while temporal attention is processed by the left parietal lobe. Further, the parietal cortex was activated bilaterally when subjects paid attention to both dimensions simultaneously. Using the same paradigm, an ERP study found that spatial attention affected the amplitude of early visual components, while temporal attention started later, and mainly affected late stages of processing related to decisions and responses (Griffin et al., 2002).

A recent fMRI study using the visual cue-auditory target paradigm reported that the dorsal frontoparietal network is involved in modulation of auditory spatial attention, and confirmed that temporal attention led to specific activation of the superior occipital gyrus, the tegmentum, the motor area, the thalamus and the putamen (Li et al., 2012). Moreover, a previous ERP study showed that the modulatory effects of visually cued spatial and temporal attention on auditory stimulus pro-cessing are different but partly overlapping (Tang et al., 2013). Specifically, the ERP results showed that spatial attention and temporal attention had different effects on P1 and late positivity, but the same effect on N1.

Furthermore, several studies have reported that visual and auditory stimuli are not processed in isolation but produce coherent cognition in the brain, indicating that responses to bimodal audiovisual stimuli (AV) are more rapid and accurate than responses to either unimodal visual (V) or unimodal auditory stimuli (A) (Gao et al., 2014; Li et al., 2010; Tang et al., 2022). This facilitative effect is called “audiovisual integration”. A study showed that bimodal stimuli can attract attention easier than unimodal stimuli (Lunn et al., 2019). And there studies indicated that the processing mode of bimodal audiovisual stimuli is different from the unimodal visual and unimodal auditory stimuli (Meredith and Stein, 1986; Tang et al., 2016). Numerous researchers have investigated the effect of visually cued spatial and temporal attention on unimodal visual or unimodal auditory stimulus processing. However, how visually cued spatial and temporal attention modulates audiovisual stimuli processing is still un-clear.

The aim of our study was to investigate how visually cued spatial and temporal attention modulate ERPs corresponding to audiovisual stimuli processing. Visual spatial cues predicted the spatial location (left, right) of stimuli, and visual temporal cues predicted a short or long time interval for cue-target processing. We utilized the high temporal resolution of ERPs to observe that stages of audiovisual stimuli processing are affected by visually cued spatial or temporal attention.

## Methods

### Participants

Eleven healthy students (age range: 21–25 years; mean age: 23.2 years; 7 male) from Okayama University participated in the experiment. All subjects were right-handed and had normal or corrected-to-normal vision and normal hearing. In addition, they had no history of neurological or psychiatric disorders. The individuals provided written informed consent for participation in this study, and the experimental protocol was approved by the Ethics Committee of Okayama University.

### Stimuli and procedures

**Figure 1:**
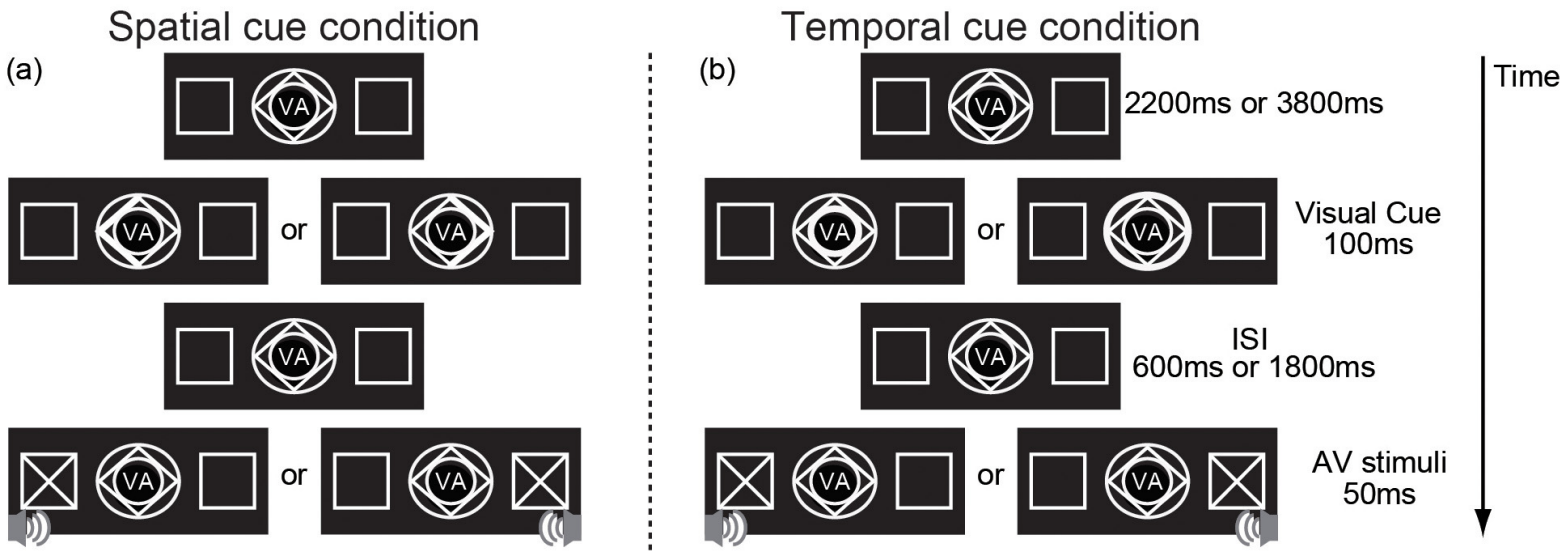
An example showing the sequence of trial events. **(a)** the visual spatial cue indicates spatial information (the left arrow indicating the left, the right arrow indicating the right) but provides no information about the cue–target interval and **(b)** indicates temporal information (the inner circle indicating the short cue–target interval, the outer circle indicating the short cue–target interval) but provides no information about the spatial information. “VA” in the center of the cue indicates that the target of this experiment is visual-auditory stimulus.

The experiment was conducted in a dimly lit, sound-attenuated, electrically shielded room. Stimulus streams were randomly presented on a black background. The participants were seated 60 cm from the center of a monitor. At the beginning of each trial, the normal stimuli, which consisted of two peripheral left and right boxes (2° × 2°, centers 7° from the center of the monitor) and fixation stimuli (2° × 2°) (see Figure 1) were presented in the center of the monitor. After 2,200 or 3,800 ms, the cue was presented for 100 ms. A spatial cue predicted the spatial location (left, right) of the audiovisual stimuli but gave no time information about the cue-target interval. Spatial cues consisted of brightening either the left or right border of a diamond to inform the subject to attend to the location at which the stimulus would be presented (left or right) (Figure 1a). The temporal cue predicted the cue-target interval but did not provide information about the location. Temporal cues were compound stimuli consisting of a diamond and two concentric circles highlighted to inform the subject to attend to the time of the cue-target interval (600 or 1,800 ms from cue presentation) (Figure 1b). If either the inner or outer border brightened, the interstimulus interval (ISI, the time interval between visual cue offset and audiovisual stimuli onset) was 600 or 1,800 ms, respectively. The AV stimuli was then presented for 50 ms. There were 2 types of AV stimuli: no-go and go stimuli. The no-go stimuli consisted of a visual stimulus (×, 2° × 2°) and an auditory stimulus (sinusoidal tone, 1,600 Hz/65 dB, with linear rise/fall times of 5 ms) presented at the same location (left or right) and accounted for 77% of the AV stimuli. No response was required to no-go stimuli. The go-stimuli consisted of a visual stimulus (+, 2° × 2°) and an auditory stimulus (similar to the no-go auditory stimulus but containing a transient dip in intensity at the halfway point of the stimulus presentation) presented at the same location and accounted for 23% of AV stimuli. The subjects were required to respond through a reaction key as accurately as possible. For each spatial cue trial, the ISI was set to either 600 or 1,800 ms randomly; for the temporal cue trials, stimuli appeared on the left or right at random.

The cue type (spatial or temporal) was blocked to evoke spatial or temporal attention independently, but the sequence was counterbalanced across participants. Participants were required to fix their eyes on a centrally presented fixation point on a screen and were allowed to take a 5-min break between blocks. There were 5 blocks per cue type. In each block, there were 40 no-go trials and 12 go trials. The total experiment time was ~2 h.

### Apparatus and software

Stimulus presentation was controlled by a personal computer running Presentation software (Neurobehavioral Systems, Albany, CA). An EEG system (BrainAmp MR plus, Gilching, Germany) was used to record EEG signals through 32 electrodes mounted on an electrode cap (Easy cap, Herrsching Breitbrunn, Germany), as specified by the International 10–20 System. All signals were referenced to bilateral earlobe electrodes. Horizontal eye movements were recorded from the outer canthus of the right eye; eye blinks and vertical eye movements were recorded from the vEOG electrode. The impedance of all of the electrodes was kept below 5 kΩ. Raw signals were digitized with a sample frequency of 500 Hz with a 60 Hz notch filter and stored continuously on a compatible computer for offline analysis. The event-related potential (ERP) analysis was carried out using Brain Vision Analyzer software (version 1.05, Brain Products GmbH, Munich, Bavaria, Germany).

### Data analysis

#### Behavioral results analysis

Reaction times (RTs) for the correct detection of go stimuli, hit rates (HRs), and false alarm rates (FARs) for incorrect responses to no-go stimuli from each participant were analyzed separately for each stimulus type. HRs were the number of correct responses to go stimuli divided by the total number of go stimuli. FARs were the number of incorrect responses to no-go stimuli divided by the total number of no-go stimuli. RTs, HRs and FARs that were different for the two cue types were subjected to a repeated-measures analysis of variance (ANOVA) using the factors of spatial cue and temporal cue conditions and a significance level of 0.05.

#### ERP results analysis

Only the ERPs elicited by the AV no-go stimuli were analyzed to remove the response movement effect. The EEG and EOG signals were amplified and band-pass filtered with an analog filter of 0.01–100 Hz at a sampling rate of 500 Hz. Continuous EEG and EOG signals were divided into epochs from 100 ms before stimulus onset to 500 ms after stimulus onset. Baseline corrections were made against −100–0 ms. Responses associated with false alarms were also rejected from the analysis. The data were then averaged for each stimulus type, following digital filtering with a band-pass filter of 0.01–30 Hz. The grand-averaged data were obtained across all participants for each stimulus type. Because no significant lateralization effect of AV stimuli has been found, ERP data from the left and right hemisphere were combined to improve the signal-to-noise ratio of the ERPs (Talsma and Woldorff, 2005). Based on the results of the analysis, the amplitudes of the N2 components were measured as the mean voltages within the intervals 300–340 ms post stimulus. The mean amplitudes were compared separately using a repeated-measures ANOVA with factors of cue type (spatial vs. temporal) and electrodes (CP2, CP6, Pz, P4, P8, O2, T8). We automatically rejected trials with vertical eye movements and eye blinks (vertical EOG amplitudes exceeding ±100 μV), horizontal eyeball movements (horizontal EOG amplitudes exceeding ±25 μV), or other artifacts (a voltage exceeding ±75 μV at any electrode location relative to baseline). The Greenhouse-Geisser epsilon correction was used for non-sphericity when appropriate. Statistical significance was set at a 0.05 level. All statistical analyses were carried out using SPSS, version 16.0 (SPSS, Tokyo, Japan).

## Results

### Behavioral results

The RTs, HRs, and FARs for each cue type condition are shown in Table 1. Significant differences between the spatial and temporal cue conditions were found for RTs and FARs. The RTs in response to go AV stimuli in the spatial cue condition were significantly faster [*F*_(1, 10)_ = 19.033, *p* < 0.001] than RTs in response to go AV stimuli in the temporal cue condition. Furthermore, the FARs for the spatial cue condition (9.5%) were significantly lower [*F*_(1, 10)_ = 7.574, *p* < 0.05] than for the temporal cue condition (16.8%). However, there were no differences in HRs [*F*_(1, 10)_ = 1.267, *p* = 0.287] associated with the spatial and temporal cue conditions. All subjects responded to the task with accuracy above 95%.

**Table 1:** Mean reaction times (RTs), hit rates (HRs), and false alarm rates (FARs) in each cue condition.

**Behavioral measures**	**Cue type**	
	**Spatial**	**Temporal**
RTs (ms)	471 (38)	583 (42)
HRs (%)	95.6 (0.9)	99.1 (0.6)
FARs (%)	9.5 (2.1)	16.8 (3.2)

Standard deviations are given in parentheses.

### ERP results

**Figure 2:**
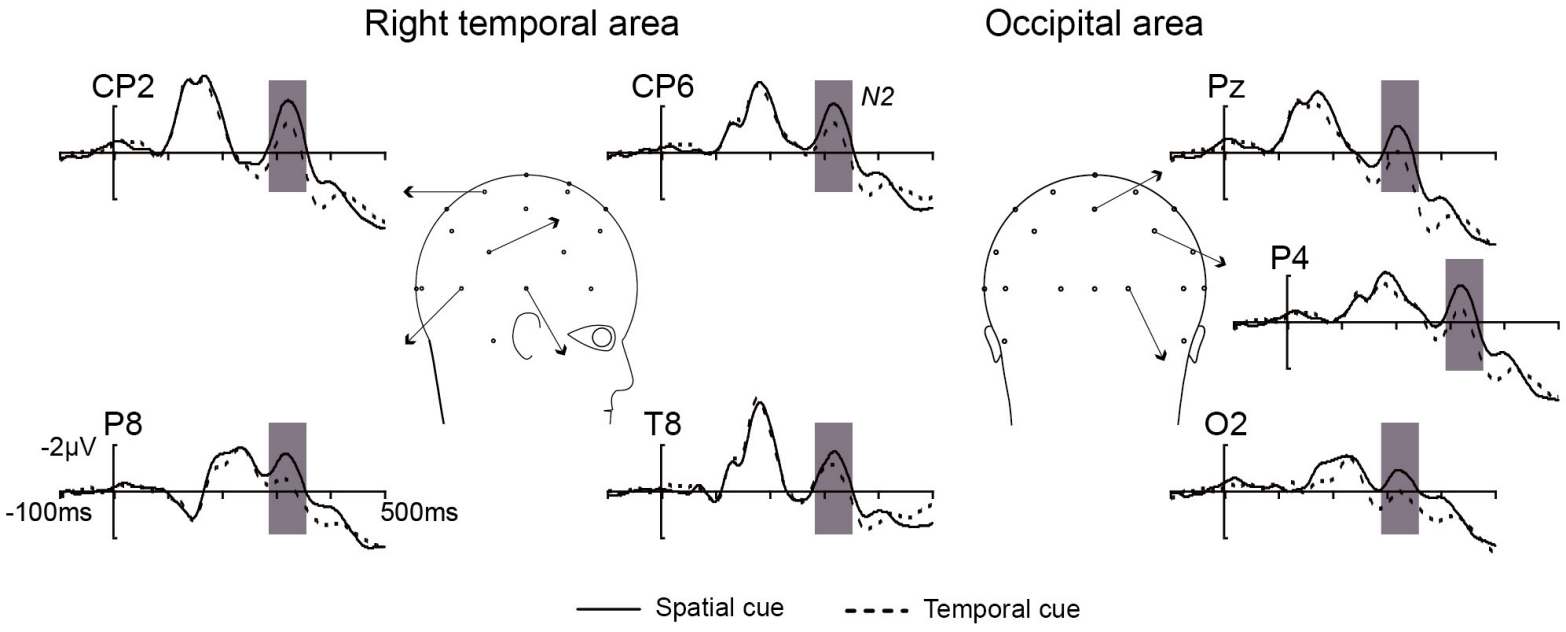
The overlap of grand average ERPs elicited by no-go AV stimuli from the right temporal and occipital electrodes in spatial cue (black solid line) and temporal cue (black dotted line) conditions.

**Figure 3:**
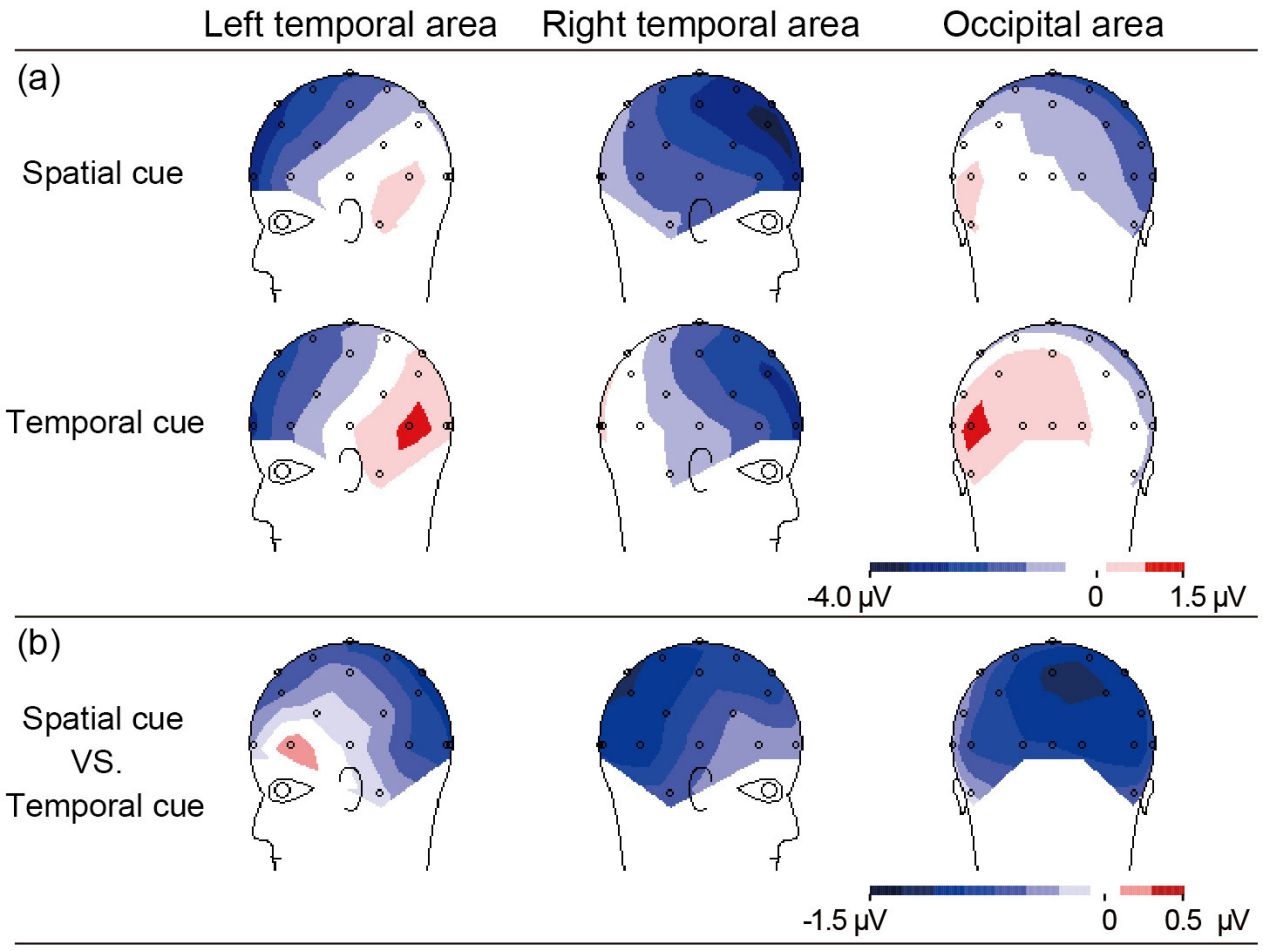
The scalp topographies of N2 activity (300–340 ms) elicited by AV stimuli at left and right temporal, occipital area related to **(a)** the effect of spatial or temporal cue, and **(b)** effect in spatial cue minus temporal cue.

As shown in Figure 2, the difference in N2 (300–340 ms) amplitude between the spatial and temporal cue condition was analyzed by ANOVA. A main effect of cue type was significant [*F*_(1, 10)_ = 11.23, *p* < 0.01] at the right temporal area and extended to the right occipital electrodes (CP2, CP6, Pz, P4, P8, O2, and T8). The result showed that the amplitude of N2 in the spatial cue condition was significantly stronger than in the temporal cue condition (Figure 2). In addition, the main effect of the electrode was significant [*F*_(6, 60)_ = 2.98, *p* < 0.05], and the interaction between the electrode and the cue type was not significant [*F*_(6, 60)_ = 1.21, *p* > 0.05]. *Post-hoc* analysis showed significant differences in amplitude at all of the calculated electrodes (*p* < 0.05), and the maximum difference was found at P4 (−1.108 μV, *p* < 0.001). The scalp distribution of the audiovisual N2 component (300–340 ms) for the spatial cues, temporal cues and the difference between the two cue types is shown in Figure 3.

## Discussion

### Behavioral data

The present study aimed to investigate differential modulation of audiovisual stimuli processing by spatial and temporal attention. We found that behavioral responses to AV stimuli in the spatial attention condition were faster than those in the temporal attention condition, which is consistent with previous studies in which responses to auditory stimuli were studied (Li et al., 2012; Tang et al., 2013). A study have proposed whereas the effects of spatial attention are evident at an early stage, the early effects of time are not easy to detect (Correa et al., 2006; Lange et al., 2006). Higher perceptual requirements may be needed to capture the effects of temporal attention (Correa et al., 2006). Our study employed a detection task with relatively low perceptual demands, which might make it more difficult to observe the facilitating effect of temporal attention, resulting in faster AV reaction times under spatial attention than under temporal attention. Our findings suggest that the AV performance in behavioral response in spatial attention are better than temporal attention.

### ERP data

We observed that a stronger N2 amplitude at the right temporal and the right occipital area was associated with a low false alarm rate in the spatial attention condition compared with the temporal attention condition (Table 1, Figure 3b). These data indicate that larger inhibitory responses were induced by spatial attention. Previous studies interpreted N2 enhancement as reflecting response inhibition when tasks required subjects to respond to a go stimulus and inhibit a response to a no-go stimulus (Nguyen et al., 2021; Jing et al., 2022).

Several neurobiological studies have investigated the effect of spatial attention (Wang et al., 1999) or temporal attention (Coull et al., 2000; Griffin et al., 2001; Miniussi et al., 1999), or the differential effects of spatial and temporal attention (Coull and Nobre, 1998; Griffin et al., 2002) on visual stimulus processing. An ERP study found that the late N2 component was larger when stimuli were presented at the attended location than when stimuli appeared at an unattended location, and this effect of spatial attention was mainly associated with the right occipitotemporal area regardless of the stimulus position (Wang et al., 1999). However, in the temporal attention condition, the N2 component was enhanced only when subjects attended to the short interval rather than the long interval (Griffin et al., 2002). In summary, an effect of spatial attention on visual N2 enhancement was found both in the left and right attended location, while the effect of temporal attention was observed only in the short interval. Participants' reaction times were found to conform to a hazard function, with reaction times speeding up as the time interval between the cue and the target increased. Once the target did not appear in the short interval, participants would redirect their attention to the long interval, and this redirection process would eliminate the influence of time attention under the long-interval condition (Capizzi et al., 2023). These findings show that enhance of the visual N2 component in amplitude during spatial attention was greater than during temporal attention. However, no difference in the amplitude of the auditory N2 component between spatial attention and temporal attention was found (Tang et al., 2013).

Furthermore, the visual and auditory information simultaneously received from different sensory modalities must be integrated by several systems to produce coherent cognition in the brain (Spence, 2007; Talsma et al., 2007). Previous studies proposed that the right temporal and the right occipital areas are the typical cortical locations of audiovisual integration in the brain, and play an important role in the integration of audiovisual information into coherent boundaries (Fort et al., 2002; Giard and Peronnet, 1999; Paulesu et al., 1995; Yang et al., 2013). Therefore, our present results may be interpreted in the differential modulation of audiovisual N2 components by visually cued spatial and temporal attention originates from the cortical area that is generally recognized as integrating audiovisual information in the brain.

## Conclusion

This study provides behavioral evidence that detection of audiovisual stimuli in spatial attention is faster than in temporal attention, and the false alarm rate for spatial attention is significantly lower than for temporal attention. Additionally, the ERP results showed that the N2 amplitude in the spatial attention condition at the right temporal and the right occipital areas is stronger than in the temporal attention condition. Our ERP results suggest that the differential modulation of the audiovisual N2 component by visually cued spatial and temporal attention was generated by an audiovisual integration region of the brain.

## Data Availability

The raw data supporting the conclusions of this article will be made available by the authors, without undue reservation.
